# Clinical outcome data of anxiety patients treated with cannabis-based medicinal products in the United Kingdom: a cohort study from the UK Medical Cannabis Registry

**DOI:** 10.1007/s00213-023-06399-3

**Published:** 2023-06-14

**Authors:** Raphael Rifkin-Zybutz, Simon Erridge, Carl Holvey, Ross Coomber, Jessica Gaffney, Will Lawn, Daniela Barros, Urmila Bhoskar, Gracia Mwimba, Kavita Praveen, Chris Symeon, Simmi Sachdeva-Mohan, James J Rucker, Mikael H Sodergren

**Affiliations:** 1 Imperial College Medical Cannabis Research Group, Department of Surgery and Cancer, Imperial College London, Academic Surgical Unit, 10th Floor QEQM, St Mary’s Hospital, South Wharf Road, London, W2 1NY UK; 2 Department of Psychological Medicine, King’s College London, London, UK; 3 Department of Psychology, IoPPN, KCL, London, UK; 4 Sapphire Medical Clinics, London, UK; 5 St. George’s Hospital NHS Trust, London, UK; 6 Clinical Psychopharmacology Unit, Department of Clinical, Educational and Health Psychology, UCL, London, UK; 7 South London & Maudsley NHS Foundation Trust, London, UK

**Keywords:** Cannabis, Cannabidiol, Tetrahydrocannabinol, Anxiety, Generalised anxiety disorder

## Abstract

**Rationale:**

Cannabis-based medicinal products (CBMPs) have been identified as novel therapeutics for generalised anxiety disorder (GAD) based on pre-clinical models; however, there is a paucity of high-quality evidence on their effectiveness and safety.

**Objectives:**

This study aimed to evaluate the clinical outcomes of patients with GAD treated with dried flower, oil-based preparations, or a combination of both CBMPs.

**Methods:**

A prospective cohort study of patients with GAD (*n* = 302) enrolled in the UK Medical Cannabis Registry prescribed oil or flower-based CBMPs was performed. Primary outcomes were changes in generalised anxiety disorder-7 (GAD-7) questionnaires at 1, 3, and 6 months compared to baseline. Secondary outcomes were single-item sleep quality scale (SQS) and health-related quality of life index (EQ-5D-5L) questionnaires at the same time points. These changes were assessed by paired *t*-tests. Adverse events were assessed in line with CTCAE (Common Terminology Criteria for Adverse Events) v4.0.

**Results:**

Improvements in anxiety, sleep quality and quality of life were observed at each time point (*p* < 0.001). Patients receiving CBMPs had improvements in GAD-7 at all time points (1 month: difference −5.3 (95% CI −4.6 to −6.1), 3 months: difference −5.5 (95% CI −4.7 to −6.4), 6 months: difference −4.5 (95% CI −3.2 to −5.7)). Thirty-nine participants (12.9%) reported 269 adverse events in the follow-up period.

**Conclusions:**

Prescription of CBMPs in those with GAD is associated with clinically significant improvements in anxiety with an acceptable safety profile in a real-world setting. Randomised trials are required as a next step to investigate the efficacy of CBMPs.

## Introduction

Anxiety disorders are the most prevalent group of mental health conditions globally affecting 301 million individuals (Yang et al. 2021). Generalised anxiety disorder (GAD) is characterised by persistent worry for at least 6 months accompanied by physical and mental symptoms of anxiety such as sleep disturbances, restlessness and muscle tension. GAD has a single-year incidence in the UK of 2.2% (Remes et al. 2018) and is an important target for treatment as it has pervasive, negative effects on quality of life (Comer et al. 2011) and is associated with increased suicidality and completed suicide (Cougle et al. 2009; Meier et al. 2016).

Current first-line pharmacological treatments for GAD in the UK are selective serotonin reuptake inhibitors (SSRIs) with other classes of medication, such as selective noradrenergic reuptake inhibitors, gabapentinoids, beta-blockers and benzodiazepines being second-line or adjunctive treatment options (Health and Excellence 2011; Taylor et al. 2021). Whilst these treatments are effective, around 50% of individuals will not respond to first-line therapy and up to 30% will fail to respond to multiple medications (Ansara 2020; Bystritsky 2006). Additionally, SSRIs can take up to 12 weeks to be effective in anxiety disorders, can cause an initial increase in anxiety and, although generally well tolerated, have a significant side effect profile which limits tolerability in some individuals (Ferguson 2001). There is therefore a need for new treatment options, especially for those who gain no benefit from current pharmaceutical options.

The endocannabinoid system has shown promise as a target for pharmacological intervention, with cannabinoid receptors, such as cannabinoid receptor type 1 (CB1R) and type 2 (CB2R) thought to be important in the anxiety response (Stampanoni Bassi et al. 2018). The phytocannabinoid cannabidiol (CBD), which is an allosteric modulator of the CB1R and enhances anandamide levels (Britch et al. 2021), has been shown in pre-clinical and clinical settings to have anxiolytic effects (Allsop et al. 2014; Bergamaschi et al. 2011; Garcia-Gutierrez et al. 2020), whereas the other prominent phytocannabinoid delta-9-tetrahydocannabinol (THC), a partial CB1R agonist, has sometimes been associated with acute increases in anxiety (Martin-Santos et al. 2012). The interaction of these two phytocannabinoids is complex, and whilst some research has shown that CBD can ameliorate the negative effects of THC, other studies have found potentiation or no effect (Englund et al. 2022; Karniol et al. 1974; Martin-Santos et al. 2012; Pennypacker et al. 2022; Sharpe et al. 2020). These studies contain heterogeneity in dose, route of administration, length of treatment and THC:CBD ratio, and the exact nature of their ratio remains to be elucidated (Freeman et al. 2019). Moreover, CBD and THC have additional off-target effects at serotonin receptors and transient receptor potential cation channel subfamily V member 1 channels, which have each been implicated in anxiety response and behaviour (Campos et al. 2012; Pertwee 2014; Rock et al. 2012). However, even these considerations simplify the complex pharmacology of cannabis, which contains over 100 phytocannabinoids whose complex interactions are not yet well understood (Sharpe et al. 2020).

Cannabis-based medicinal products (CBMPs) were made available for specialist medical prescription in the UK from 2018. The UK Medical Cannabis Registry, set up in December 2019, is the first prospective registry that records the pseudonymised data of patients prescribed CBMPs, across the UK and Channel Islands, and is managed by Sapphire Medical Clinics (Erridge et al. 2021). This registry offers the opportunity to examine the effects of cannabis within patients prescribed products which meet Good Manufacturing Practice criteria.

An earlier paper that investigated 67 patients with GAD enrolled on the UK Medical Cannabis Registry showed improvements in anxiety and few safety concerns. Whilst results at 1 month showed an average reduction in 4.5 points on the GAD-7, there was a paucity in longer term outcome data with only 13 patients with data at 6 months follow-up (Ergisi et al. 2022). The UK Medical Cannabis Registry has now been running for longer and enrolled many more patients which allows for better analysis of longer term data, including the original cohort.

This updated analysis from the UK Medical Cannabis Registry aimed to study a larger cohort of patients treated with CBMPs for the principal indication of GAD. The primary aim was to assess changes in anxiety symptoms as measured by the GAD-7 and the incidence of adverse events as collected by self-report form. The effect of type of CBMP product or previous cannabis experience influenced GAD-7 scores or the reporting of an adverse event was also analysed. The secondary aim was to investigate sleep quality and health-related quality of life outcomes.

## Methods

### Study design and participants

The reporting of this observational study conformed to STROBE guidelines (von Elm et al. 2008). Formal ethical approval for the UK Medical Cannabis Registry has been provided by the Health Research Authority (South West—Central Bristol Research Ethics Committee reference 22/SW/0145). Written and informed consent was completed by all participants prior to enrolment.

Participants who gave consent and were prescribed CBMPs were enrolled consecutively into the UK Medical Cannabis Registry. Exclusion criteria for this study included incomplete baseline recording of the generalised anxiety disorder-7 (GAD-7) scale and enrolment for less than 1 month.

In this cohort study, data were extracted from the UK Medical Cannabis Registry for patients prescribed CBMPs for GAD as the primary indication for treatment. In the UK, CBMPs may only be prescribed for individuals with conditions that have not been satisfactorily relieved by licensed therapies (Case 2020). Consequently, prior to enrolment, all patients had already received a diagnosis of GAD and undertaken an adequate trial of pharmacological or psychological therapies, as appropriate. The CBMPs prescribed were produced in line with Good Manufacturing Practice and were prescribed by a specialist in the condition, with the decision ratified by a multidisciplinary team, as per national guidance (Regulatory Advice Unit 2020). There was no requirement for a washout period prior to initiation of treatment in those individuals already consuming cannabis obtained without prescription. However, participants are counselled against continued use of illicit cannabis.

### CBMP details

The formulations were either dry flower (flos or granulate) or oil (isolate phytocannabinoids or full-spectrum products containing cannabinoids, terpenes and flavonoids). The oils were administered orally or sublingually, whilst dry flower formulations were inhaled. Inhalation involves the use of a vaporisation device that heats the dry plant to release the active pharmaceutical ingredients, which can be inhaled by the patient (Chaiton et al. 2022). Furthermore, the strains were either *Cannabis sativa*, *Cannabis indica*, or a hybrid species. Details of individual CBMP prescriptions were recorded at baseline and throughout treatment including formulation and CBD and THC dose per day (mg).

### Data collection

The following demographic data were collected at the initial assessment: age, gender, occupation and body mass index (kg/m^2^). The primary indication for treatment with CBMPs, other indications where applicable and comorbidities were recorded. The Charlson comorbidity index, a prognostic tool used to predict the mortality of patients for external benchmarking against other patient cohorts, was calculated for each participant (Quan et al. 2011).

Data were collected remotely whereby patients received patient-reported outcome measures (PROMs) and adverse event questionnaires electronically via an online web-based platform at baseline and 1 month, 3 month and 6 month follow-ups (Tait et al. 2023). Participants could complete PROMs even if they had not completed those in prior periods, except in instances where the baseline PROMs were incomplete. Patients join the clinic continuously and so some had not yet had the time elapse to some of the measured time points. Missing data was therefore worked out as the proportion of those without coded responses out of those who had their baseline assessment at least 1, 3 or 6 months prior. Missingness was defined as no data for a participant who had been in the cohort for long enough to expect a follow-up to have taken place.

Information on prior tobacco, alcohol and illicit cannabis use was collected and quantified using pack years, weekly alcohol consumption in units and current quantity of cannabis consumption in grams per day respectively. To quantify the individual history of using cannabis, a novel metric of ‘cannabis gram years’ was utilised, as previously described by our group (Erridge et al. 2021). Recording of cannabis consumption incorporated use of all illicit and legal products obtained either in the UK or when in another jurisdiction. Harmful alcohol use was coded as a binary variable, defined as greater than 14 units consumed a week in accordance with national UK guidelines (Health 2016).

Other medications under the following classes were also recorded: analgesics, anticoagulants, antidepressants, antidiabetic drugs, antimigraine drugs, antiplatelets, hypnotics and anxiolytics.

### Patient-reported outcomes

As the cohort was being treated for GAD, change in severity of symptoms was considered the outcome of primary interest. GAD-7 is a validated self-reported questionnaire designed to screen and measure severity for GAD (Spitzer et al. 2006). Subjects are asked about the seven core symptoms of GAD over the last 2 weeks. The total score is from 0 to 21, with thresholds of ≥5, ≥10 and ≥15 signifying mild, moderate and severe anxiety symptoms, respectively. Response was defined as a reduction in anxiety scores by at least 50% (Bandelow 2006). The minimally clinically importance difference was defined as an absolute change of 4 points on the GAD-7 scale, regardless of baseline anxiety score (Toussaint et al. 2020).

The sleep quality scale (SQS) is a validated single-item question to assess overall sleep quality in the last 7 days (Snyder et al. 2018). A self-reported scale of 0–10 is used, and the following sleep quality categories are formed: terrible (0), poor (1–3), fair (4–6), good (7–9) and excellent (10) (Snyder et al. 2018).

The EQ-5D-5L is a self-reported and validated questionnaire measuring health-related quality of life (Herdman et al. 2011). Subjects are asked to rate their quality of life on the day of completing the questionnaire across five domains: ‘mobility’, ‘self-care’, ‘usual activities’, ‘pain/discomfort’ and ‘anxiety/depression’. The resulting health state is mapped to index values validated for a UK population (van Hout et al. 2012). Optimum health is given an index score of 1, whilst a negative index value represents a perceived health state worse than death.

Adverse events were collected at each follow-up interval through self-reporting, routine follow-up with a clinician or direct questioning by the research team. These events, their length in days and their severity were recorded in accordance with the Common Terminology Criteria for Adverse Events v4.0 (CTCAE) (Trotti et al. 2003). The CTCAE allows for coding of each adverse event and rates severity on a 5-point scale from mild to death due to adverse event.

### Statistical analysis

Baseline information was analysed using descriptive statistics. Differential missingness across groups at months 1, 3 and 6 was assessed using a multivariate logistic regression. Baseline anxiety, type of CBMP prescribed at baseline, age, gender, cannabis use history, smoking use history, harmful alcohol use and Charlson comorbidity index were assessed for association with missingness.

Statistical significance in the change in PROMS from baseline was calculated for each time point separately using paired *t*-tests. Within each PROM, results were corrected for multiple comparisons using the Bonferroni-Holm correction for multiple comparisons (Holm 1979).

Clinical significance in the change in PROM score was evaluated by reference to previously described minimally clinically significant changes. GAD-7 has been shown to be sensitive to change with a minimally clinically significant difference of 4 (Toussaint et al. 2020). Likewise, although there are no direct studies in GAD, in other anxiety conditions such as PTSD (Le et al. 2013), the EQ5D minimally clinically significant difference was previously reported as 0.07. The initial validation of SQS suggested an improvement of 2.6 corresponded to ‘somewhat improved sleep’ which was taken as a measure of clinical significance (Snyder et al. 2018). Response rates for GAD-7 were identified as a 50% reduction in symptoms from baseline.

Factors affecting the change in anxiety, sleep and quality of life scores were assessed by a mixed linear model. Factors of interest were prior cannabis experience and type of product used. As above, the models also controlled for confounding factors, specifically baseline anxiety, age, gender, smoking use history, harmful alcohol use and Charlson comorbidity index.

Adverse events were summarised using descriptive statistics. In the summary tables, each individual’s most severe report of any particular side effect was reported as their side effect severity. To assess if any baseline values were associated with an increased chance of reporting an adverse event, a logistic regression was conducted for report of any adverse events with baseline anxiety, type of CBMP prescribed at baseline (oil, flower or both), age, gender, cannabis use history, smoking use history, harmful alcohol use and Charlson comorbidity index as possible factors.

Data processing and statistical analysis were performed with Python V3 and Stata 17.0 (Foundation 2022; StataCorp 2022).

### Sensitivity analyses

As some patients switched the form of CBMP they were using during the 6 months and the analysis used the formulation prescribed at baseline, a sensitivity analysis was performed examining factors affecting anxiety response, excluding those who switched product use during the 6 months. An additional sensitivity analysis was performed to assess the effect of being prescribed antidepressant medication at baseline.

## Results

### Baseline demographics and missing data

**Fig. 1:**
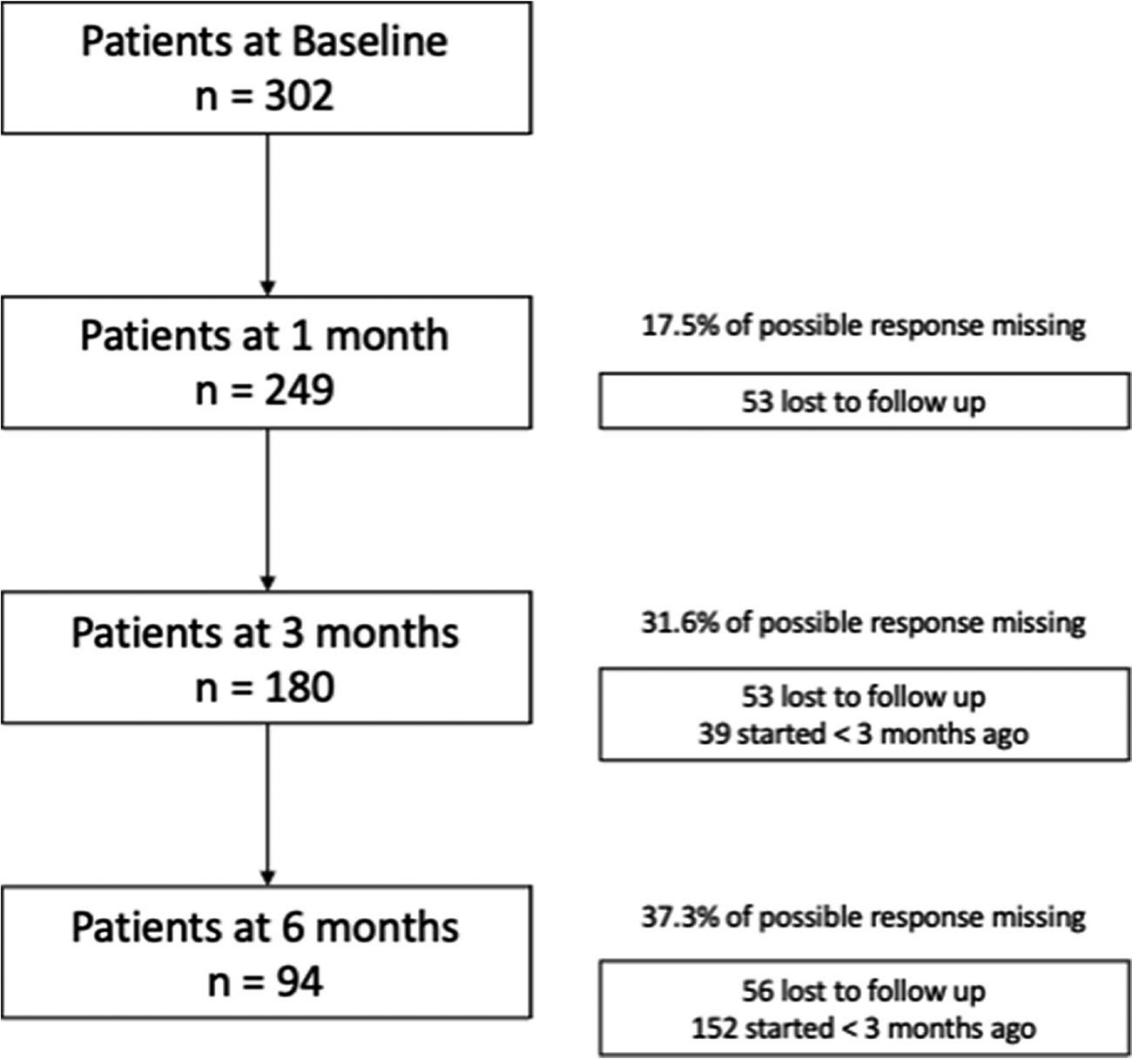
Flow diagram of participant progress through follow-up. Participant number lost to follow-up and reporting of participants who had not yet been enrolled for long enough in the UK Medical Cannabis Registry to reach that follow-up period are reported in comparison to baseline, rather than previous follow-up

Three hundred two patients had baseline anxiety assessments and were included in the analysis. There were moderate rates of missing data with 17.5% missing at 1 month (53/302), 31.6% at 3 months (83/263) and 37.3% at 6 months (56/150) (Fig. 1).

The cohort was majority male (*n* = 207, 68.6%), with a varied age range (37.0 years, SD 11.5) with a majority having either prior or current experience of cannabis use (*n* = 260, 86.1%). Approximately half the cohort (*n* = 161, 53.3%) was on antidepressants when starting CBMPs. The median participant last had a change in their antidepressant prescription 339 days ago (IQR: 80–942 days). The median anxiety level at baseline was 14.0 (IQR: 9.0–18.0) which is in the upper range of moderate anxiety as measured by the GAD-7. The most commonly prescribed products were Adven^®^ 0% CBD/20% THC (hybrid) flos (380 prescriptions), Adven^®^ 50mg/ml CBD broad spectrum oil (159 prescriptions) and Adven^®^ 20mg/ml THC full spectrum (Hybrid/indica) oil (152 prescriptions). Full baseline information is found in Table 1.

**Table 1:** Baseline characteristics of participants and prescribed cannabis-based medicinal products

			Missing
Age in years	Mean (sd)	37.0 years (11.5)	*N* = 0
BMI	Mean (sd)	26.9 kg/m^2^ (7.4)	*N* = 22
Charlson comorbidity index	Median (IQR)	0 (0 to 0)	*N* = 0
		*N* (%)	
Gender	Male	207 (68.6%)	*N* = 0
	Female	95 (31.5%)	
Tobacco use	Current	117 (38.7%)	*N* = 0
	Previous	114 (37.8%)	
	Never	71 (23.5%)	
Cannabis use	Current	188 (62.3%)	*N* = 0
	Previous	72 (23.8%)	
	Never	42 (13.9%)	
Alcohol use	Yes	164 (54.3%)	*N* = 0
	No	138 (45.7%)	
Concurrent medication	Any antidepressant	161 (53.3%)	*N* = 0
	SSRI	111 (36.7%)	
	Benzodiazepine or Z-drug	61 (20.2%)	
	Gabapentinoid	19 (6.3%)	
	Beta-blocker	33 (10.9%)	
Substance use frequency (in current users only)			
Cannabis gram per day use	Median (IQR)	1.0 (0.5 to 2.0)	*N* = 0
Alcohol intake in units weekly	Median (IQR)	0.0 (0.0 to 6.0)	*N* = 0
Smoking pack years	Median (IQR)	10.0 (2.0 to 21.0)	*N* = 0
Psychometric questionnaire baseline scores			
GAD-7	Median (IQR)	14.0 (9.0 to 18.0)	*N* = 0
SQS	Median (IQR)	3.0 (2.0 to 6.0)	*N* = 1
EQ5D-5L index value	Median (IQR)	0.58 (0.32 to 0.74)	*N* = 1
Cannabis-based medicinal products used in cohort			
CBMP	Oil	89 (29.5%)	*N* = 0
	Flower	139 (46.0%)	
	Both	74 (24.5%)	
Daily CBD dose at baseline	Median (IQR)	2.0 mg (0.1 to 20 mg)	
Daily THC dose at baseline	Median (IQR)	21.0 mg (19.0 to 40.0 mg)	

*CBD*, cannabidiol; *CBMP*, cannabis-based medicinal product; *IQR*, interquartile range; *sd*, standard deviation; *THC*, delta-9-tetrahydocannabinol

### Cannabis-based medicinal products

Patients received a variety of formulations of CBMP at their first visit with 89 (29.5%) receiving an oil-based product, 139 (46.0%) receiving a flower-based product and 74 (24.5%) being prescribed both. The type of product prescribed differed according to prior cannabis consumption (*X*^2^ = 54.4, *p* < 0.001) with cannabis-naïve patients more likely to be prescribed oil-based products, whilst prior users were evenly split between oil and flower products and current users favoured flower-based products (Table 2).

**Table 2:** Initial prescriptions of CBMPs according to prior cannabis use

Type of product	Prior cannabis use			
	Current	Past	Never	Total
Flower	102	27	9	138
Oil	31	28	30	89
Combined	54	17	3	74
Total	187	72	42	301

*X*^2^ = 54.4, *p* < 0.001

Logistic regressions were fitted to estimate if any baseline demographic factors influenced the probability of missingness. There were no factors associated with missingness at 1 month. Age was associated with missingness at 3 months (odds ratio per year older 0.96, CI 0.93 to 1.00, *p* = 0.033) and harmful alcohol use at 6 months (odds ratio 2.98, 95% CI 1.08 to 8.24, *p* = 0.036). These results are statistically insignificant after Bonferroni-Holm adjustment for multiple comparisons (8 baseline factors).

### Anxiety, sleep and health-related quality of life changes

**Fig. 2:**
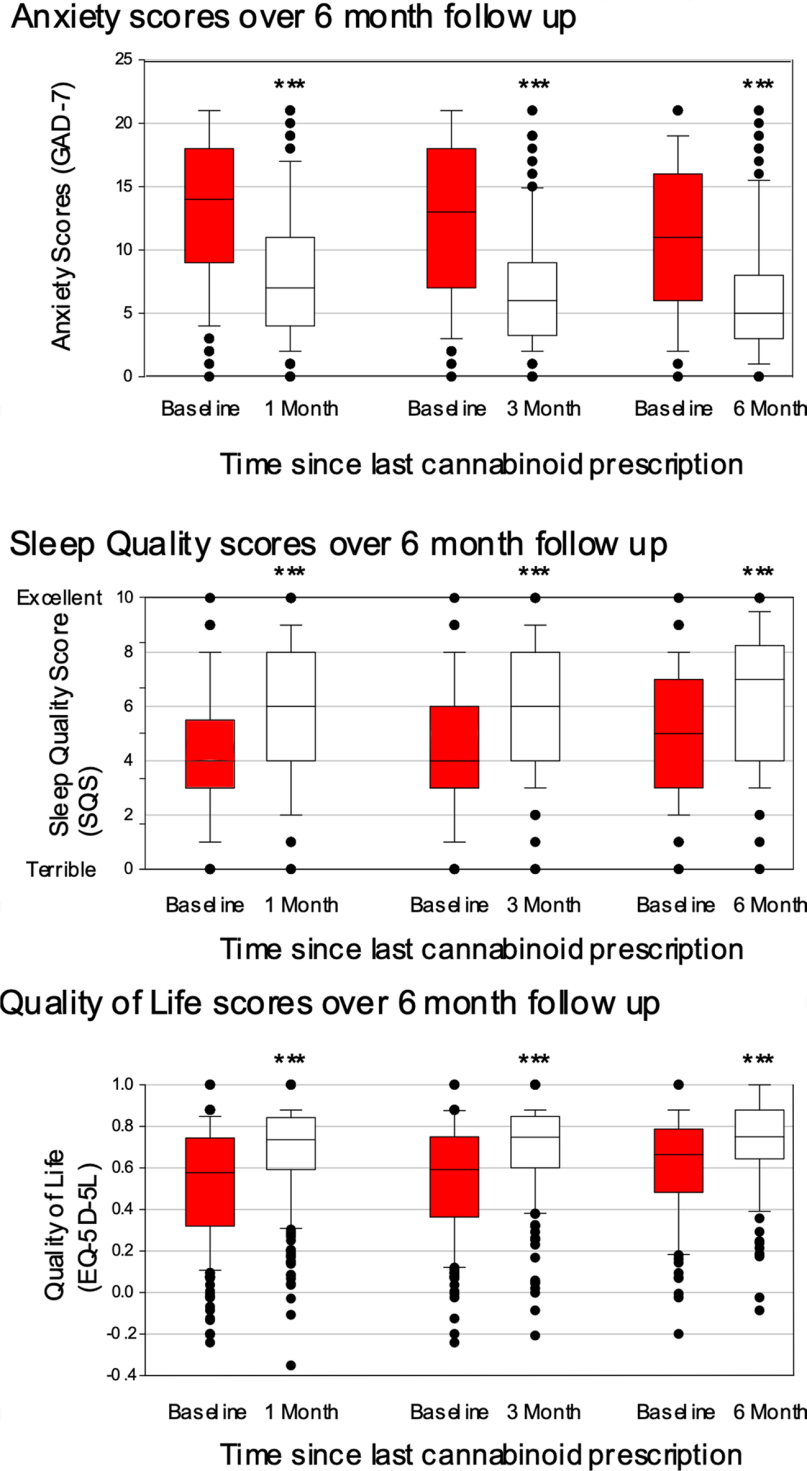
Scores of the patient-reported outcome measures at all follow-up points. The boxes represent the median and interquartile range, whilst the whiskers represent the minimum and maximum values. Paired *t*-tests were performed to test for statistical significance between the follow-up and baseline scores. For each comparison, baseline scores are for those who completed follow-up to that time point, ***p ≤ 0.001

Paired *t*-tests were used to compare patients scores at 1, 3 and 6 months compared to baseline. Patients receiving CBMPs had improvements in GAD-7 at all time points (1 month: difference −5.3 (95% CI −4.6 to −6.1), 3 months: difference −5.5 (95% CI −4.7 to −6.4), 6 months: difference −4.5 (95% CI −3.2 to −5.7), all *p* < 0.001) (Fig. 2).

GAD-7 response rates (50% or greater reduction in scores) were 38.2% (95/249) at 1 month, 40.1% (73/180) at 3 months and 38.3% (36/94) at 6 months. Minimally clinically significant improvements of a 4-point decrease in GAD-7 scores were seen in 58.2% (145/249) participants at 1 month, 55% (99/180) of participants at 3 months and 43.6% (41/94) participants at 6 months.

Improvements in sleep scores were seen at all time points (1 month: difference 1.8 (95% CI 1.5 to 2.2), 3 months: difference 1.9 (95% CI 1.6 to 2.3), 6 months: difference 1.5 (95% CI 0.9 to 2), all *p* < 0.001). Improvements in quality of life scores were seen at all time points (1 month: difference 0.15 (95% CI 0.12 to 0.18), 3 months: difference 0.15 (95% CI 0.11 to 0.18), 6 months: difference 0.11 (95% CI 0.06 to 0.16), all *p* < 0.001) (Fig. 2).

These results are significant after Bonferroni-Holm correction for multiple comparisons.

### Predictive factors

To investigate possible factors that were predictive of the effects of CBMPs, a linear mixed model was fitted to assess the influence of CBMP used and prior cannabis experience on anxiety levels while taking CBMPs. Prior cannabis experience (group × time, *X*^2^ = 8.27, *p* = 0.22) and product prescribed at baseline (group × time, *X*^2^ = 10.7, *p* = 0.22) were not associated with differential changes in anxiety.

### Sensitivity analysis

Analyses investigating factors influencing response were rerun excluding those who switched medications. These were substantively the same with neither product use at baseline (group # time, *X*^2^ = 12.1, *p* = 0.15) nor prior cannabis experience (group # time, *X*^2^ = 5.8, *p* = 0.45) associated with changes in anxiety over time.

Anxiety levels were not significantly different at baseline for those on an antidepressant compared to those who were not (difference 0.34 (−1.14 to 2.42), *p* = 0.71). Antidepressant use (group × time, *X*^2^ = 2.02, *p* = 0.57) was not associated with differential changes in anxiety.

### Adverse events

Thirty-nine participants (12.9%) reported any adverse events, with 269 adverse events reported across these 39 participants. Eleven participants (3.6%) reported at least one adverse effect at a severe intensity. The most common adverse effects were dry mouth (*n*=25; 8.3%), fatigue (*n*=22; 7.3%), insomnia (*n*=19; 6.3%), somnolence (*n*=16; 5.3%), lethargy (*n*=16; 5.3%) and nausea (*n*=16; 5.3%). The most reported severe adverse effect was insomnia with 6 (1.99%) participants rating their insomnia as severe. No side effects were reported as life-threatening/disabling. A full list of side effect severities for side effects reported by five or more individuals is found in Table 3.

**Table 3:** Reported adverse events by patients

Side effect	Mild	Moderate	Severe	Total
Dry mouth	20	5	0	25 (8.3%)
Fatigue	9	10	3	22 (7.3%)
Insomnia	6	7	6	19 (6.3%)
Somnolence	0	14	2	16 (5.3%)
Lethargy	7	8	1	16 (5.3%)
Nausea	11	3	2	16 (5.3%)
Concentration impairment	10	4	1	15 (5.0%)
Headache	9	4	2	15 (5.0%)
Confusion	10	2	1	13 (4.3%)
Dizziness	3	4	3	10 (3.3%)
Cognitive disturbance	5	3	2	10 (3.3%)
Delirium	5	4	0	9 (3.0%)
Decreased weight	9	0	0	9 (3.0%)
Amnesia	5	2	2	9 (3.0%)
Vertigo	4	3	0	7 (2.3%)
Ataxia	4	3	0	7 (2.3%)
Upper abdominal pain	4	2	0	6 (2.0%)
Anorexia	2	2	2	6 (2.0%)
Constipation	4	1	0	5 (1.7%)
Dysgeusia	2	2	1	5 (1.7%)
Anxiety	1	1	3	5 (1.7%)
Tremor	3	2	0	5 (1.7%)
Pharyngitis	0	5	0	5 (1.7%)
Muscular weakness	2	2	1	5 (1.7%)
Vomiting	4	0	1	5 (1.7%)

A logistic regression investigating factors associated with reporting any adverse effects found no statistically significant results with respect to baseline demographics, although prior cannabis experience has a trend to significance (*X*^2^ = 5.9, *p* = 0.054), with those who had never used cannabis being more likely to report adverse events than current users (odds ratio 3.42 (95% CI 1.24 to 9.43)).

## Discussion

This prospective, observational study investigated outcome data from a clinical registry of patients prescribed CBMPs for the treatment of GAD in a large medical cannabis clinic based in the UK. Results suggest that the use of CBMPs was associated with significantly improved anxiety, sleep and quality of life measures at 1, 3 and 6 months after prescription.

The mean absolute reductions at 6 months of 4.5 points in GAD and increase of 0.11 in quality of life are above levels taken at minimal clinically significant differences (Toussaint et al. 2020) (Le et al. 2013), while the average increase in sleep score of 1.5 is slightly below the level of 2.6 corresponding to ‘somewhat improved sleep’ (Snyder et al. 2018). Individually at 6 months, 39% of individuals experience a clinically significant improvement in their anxiety, 50% in their quality of life and 35% in their sleep score. These results add weight to a previous analysis (Ergisi et al. 2022), with a larger group of 96 patients followed up for 6 months compared to only thirteen previously. This suggests the improvements seen with CBMPs in this patient group are sustained across multiple domains over 6 months.

The proportipon of patients experiencing adverse events reported here (12.9%) is in line with the wider literature where rates of around 10% are common and severe side effects were rare (Gulbransen et al. 2020). The finding that those with no prior cannabis experience are more likely to report side effects than those currently using cannabis is unsurprising and likely reflects a lower level of tolerance and experience with the drug. Cannabis-based products seem to have a different profile of side effects to SSRIs with more prominent lethargy but a notable absence of sexual side effects, with only a single person reporting mild loss of libido.

The cohort in this study is unlikely to be representative of the average population of patients with GAD, given the high use of non-medical cannabis prior to enrolment (62.3%) and a disproportionate number of males (68.6%). Additionally, at baseline, around a third of the cohort had a GAD-7 score below 10, the typical screening cut-off score, and more than 50% were already on antidepressants (Spitzer et al. 2006). This suggests that a large proportion of patients might be seeking treatment for remnant symptoms persistent on other treatments. This may reduce the number of patients experiencing clinically significant reductions in anxiety due to a floor effect.

This is a unique cohort of patients and its size and length of follow-up of patients treated with cannabis offer a unique chance to study the real-world impact of medicinal cannabis. As a cohort study, it does not contain randomisation or placebo comparators and is subject to several sources of bias including expectation, regression to the mean, unidentified confounders and moderate levels of missing data.

Beyond the limitations that affect all observational studies, this study is subject to specific confounders. Over 85% of patients had previously consumed or were consuming non-pharmaceutical grade cannabis products at the point of enrolment. This introduces a significant selection bias. Many of these individuals may have accessed illicit products for the purpose of self-medicating their health condition and then decided to pursue a prescription due to the known effects. The lack of a washout period, whilst making it difficult to standardise treatment protocols across all 302 participants, does provide interesting evidence to the supplementary associated benefits reported by individuals after accessing CBMPs. It is well-documented that habitual cannabis consumers develop tolerance to the acute effects of cannabis (Colizzi and Bhattacharyya 2018). Therefore, these results suggest supplemental benefits of transitioning to pharmaceutical grade CBMPs, such as consistency of quality, medical oversight, lower exposure to illegal activities and absence of potentially harmful contaminants (Case 2020; Dryburgh et al. 2018). Whilst it is not possible to determine whether patients subsequently abstained from all other forms of cannabis after initiating therapy with CBMPs, data from UK patients does suggest that there is a reduction in illicit cannabis consumption for both recreational use and to self-treat health conditions (Troup et al. 2022). It could be argued that participants also have an expectancy bias due to positive coverage of the effects of CBMPs (Gedin 2022 #130). This could be further exacerbated due to unlicensed CBMPs being exclusively available on a private prescription which is unusual within the context of UK healthcare. Finally, CBMPs are believed to cause an exaggerated placebo response in patients due to the psychoactive and vasoactive effects of THC in particular (Gertsch 2018 #131).

These limitations clearly demonstrate the need for randomised controlled trials in the setting of GAD. However, this cohort demonstrates reported improvements in anxiety symptoms in an open real-world setting with significant benefits for patients on self-reported anxiety, quality of life and sleep and a favourable safety profile. This suggests that the next stage in determining efficacy should be a randomised placebo-controlled trial of CBMPs to investigate their effect on reducing anxiety in generalised anxiety disorder.

## Conclusion

This cohort study demonstrates that prescription of CBMPs in those with GAD is associated with clinically significant improvements in anxiety with an acceptable safety profile. Placebo-controlled randomised controlled trials are needed as the next step to test the efficacy of CBMPs in treatment of GAD.

## Data Availability

Restrictions exist on distribution of data. For availability, please contact the corresponding author directly.
